# Tobacco use behaviors in response to menthol restriction: A scoping review

**DOI:** 10.18332/tid/200694

**Published:** 2025-02-28

**Authors:** Esme E. Wright, Emanuel Tewolde, Ahmad El-Hellani, Min-Ae Song

**Affiliations:** 1 Division of Environmental Health Sciences, College of Public Health, The Ohio State University, Columbus, United States; 2 Center for Tobacco Research, The Ohio State University Comprehensive Cancer Center, Columbus, United States

**Keywords:** menthol ban, switching, quitting, behavior responses

## Abstract

**INTRODUCTION:**

Understanding how menthol smokers change their behaviors in response to a menthol ban is important for public health and tobacco control. The goal of this scoping review is to summarize the up-to-date literature on this topic.

**METHODS:**

On 9 January 2024, we searched PubMed using the terms ‘menthol ban and responses’, ‘menthol ban and quitting’, and ‘menthol ban switching’, and performed forward citation tracking of recent review articles. We extracted data from each study regarding: 1) target population (US vs non-US); 2) type of ban (hypothetical or actual menthol ban); and 3) behavioral responses, including intended outcomes (quitting), harm reduction options (switching to e-cigarettes), and unintended consequences (continuing or switching to non-menthol products).

**RESULTS:**

Our search resulted in 25 publications, including hypothetical bans (n=15), actual bans (n=6), and both scenarios (n=4); 95% and 73% of publications reported more than one behavior change under hypothetical and actual menthol bans, respectively. The majority of the US studies reported predicted behavior transitions under hypothetical bans (89%), while non-US studies have focused on actual menthol bans (73%).

**CONCLUSIONS:**

Generally, the reported behavior transitions under hypothetical and actual bans largely vary in the US and non-US, identifying research gaps regarding geographical coverage, age-specific considerations, and racial/ethnic representation. This scoping review highlights a future research agenda to encourage the public health research community to collect historical data before and after a federal menthol ban.

## INTRODUCTION

The evidence is compelling, and the need is critical: menthol cigarettes should be banned to protect public health in the United States (US)^1-5^. Nonetheless, based on up-to-date publications, this scoping review aims to highlight the trends of tobacco use behaviors among menthol smokers in response to actual menthol bans (e.g. in the EU, Canada, California, and Massachusetts) or hypothetical bans in the US and other jurisdictions.

A recent systematic review conducted a meta-analysis of literature published until 2022 on menthol cigarette bans based on 16 publications relevant to the behavior impacts of hypothetical and actual menthol bans. The authors concluded that banning menthol cigarettes could promote smoking cessation among menthol cigarette users, positively impacting public health^6^. Empirical studies from jurisdictions with actual menthol bans and modeling simulations of potential bans agreed on rates of quitting and switching to other tobacco products among menthol smokers.

To support the US Congress passing a federal menthol ban, it is necessary to present up-to-date data to reinforce the case for the FDA’s rule and highlight the potential of a federal menthol ban to promote public health in the US. Thus, our goal in this scoping review is to include up-to-date publications until 2024, to capture more comprehensive evidence by including recent evidence collected from actual bans from the US after the Food and Drug Administration announced a proposed rule to ban menthol as the last characterizing flavor in cigarettes^7^. Further, to deliver current evidence more informatively, we categorized the findings into target population (US vs non-US) and type of ban (hypothetical or actual menthol ban). We further categorized behavioral responses into intended outcomes (quitting), harm reduction options (switching to e-cigarettes), and unintended consequences (continuing or switching to non-menthol products). Additionally, we end this scoping review with a research agenda to encourage the public health research community to collect historical data before and after a federal menthol ban. We also highlight the importance of ‘targeting’ subpopulations with communication campaigns and interventions to counter the targeting of these groups by the tobacco industry to promote a more just and equitable public health in the US. We conclude by highlighting the potential of a federal ban on menthol cigarettes to benefit public health in the US by reducing smoking in general and addressing longstanding tobacco-related health disparities, particularly among racial minorities.

## METHODS

To identify relevant peer-reviewed publications on actual and hypothetical menthol bans and behavior responses of menthol cigarette smokers, a scoping review of the literature following the PRISMA extension for Scoping Reviews reporting guidance^8^ was conducted.

### Eligibility criteria

Research articles were retained if they were in English and reported behavior transition scenarios under hypothetical and actual menthol bans.

### Information sources and data items

On 9 January 2024, we searched PubMed using the search terms: ‘menthol ban and responses’, ‘menthol ban and quitting’, and ‘menthol ban switching’. Additionally, we performed forward citation tracking of recent review articles^6,9^ to capture relevant articles.

### Selection process and data collection process

The list of articles identified in PubMed was uploaded to Covidence to remove duplicate records. A reviewer (EW) excluded non-original research articles and added articles from forward citation tracking. The reviewer conducted a full-text assessment and documented the study type (actual vs hypothetical ban), title, first authors, year of publication, PMID, country, the number of participants, age, and the rates of behavior transitions, in a Microsoft Excel spreadsheet. The articles were then categorized on the following main factors: 1) Jurisdiction (US vs non-US); 2) type of ban (hypothetical or actual implemented menthol ban); and 3) behavioral responses, including quitting, switching to reduced-risk products, or continued menthol or other forms of smoking. The rates of behavior transition for each category were also documented. An additional full-text assessment was independently conducted to ensure the main factors and rates of behavior transitions by two reviewers (ET and MAS). Any disagreement was discussed between the reviewers until a consensus was reached.

### Data summary

To synthesize the transition rates of behaviors for each category, we presented median values of behavior changes along with their minimum and maximum rates from the identified studies for the overall summary. We used a pie chart to plot a proportion of identified papers from US and non-US studies and a harvest chart to display the number of publications by country (US vs non-US) across years of publication and age groups under hypothetical and actual bans.

## RESULTS

**Figure 1:**
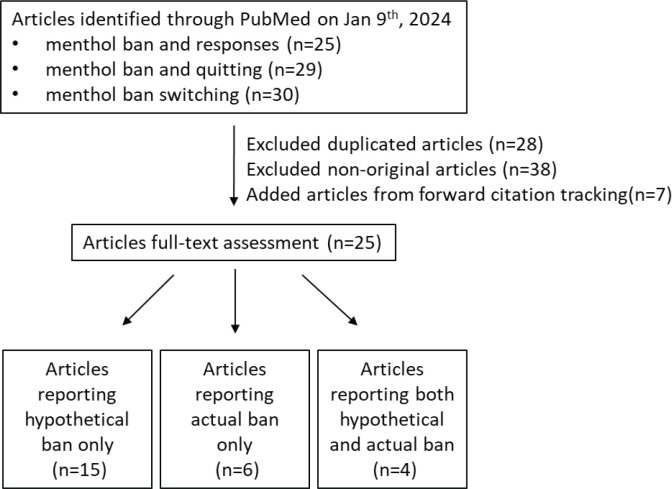
A flow chart diagram of the scoping review regarding behavior transitions under hypothetical and actual menthol bans

**Figure 2:**
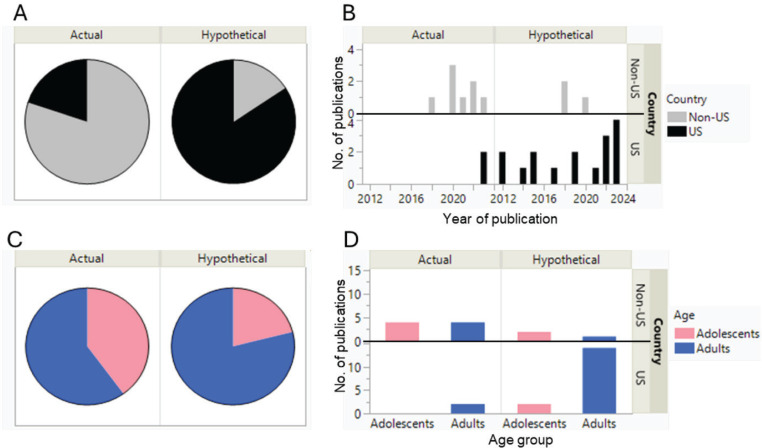
The number of publications based on actual and hypothetical bans, years of publications, and age categories in US and non-US studies: A) In the US (black) and non-US studies (grey); B) Across years in the US (black) and non-US studies (grey); C) Conducted in adolescents (pink, <18 years) and adults (blue, ≥18 years); D) Conducted in adolescents (pink, <18 years) and adults (blue, ≥18 years) in the US and non-US studies

A total of 25 publications, including studies on actual bans (n=6), hypothetical bans (n=15), and both scenarios (n=4), were considered for this scoping review (Figure 1, Table 1). Table 1 shows the distribution of the collected literature based on geographical location, type of menthol ban, and the intended behaviors covered in the study. There were more actual ban studies outside the US [73% (8/11) vs 11% (2/18) in the US]. Only 21% (4/19) and 40% (4/10) of the studies included adolescents in their study population under hypothetical and actual bans, respectively; 95% (1/18) and 73% (3/11) of the publications reported more than one behavior change under hypothetical and actual menthol bans, respectively (Table 2, Figures 2A and 2B). In the US studies, all studies (n=16; 100%) reported quit rates, but there has been less attention to capturing continued menthol smoking (50%, 8/16) under a hypothetical ban scenario. The studies on behavior changes following menthol bans primarily focused on individuals aged ≥18 years, with 79% (15/19) and 60% (6/10) of the participants falling into this category in hypothetical and actual ban studies, respectively (Figure 2C). Neither recent US study under actual bans included adolescents (Figure 2D).

**Table 1:** List of publications included in scoping review

*Bans*	*Authors Year*	*PMID*	*Journal*	*Country*	*Race/Ethnicity*	*Participants*	Age group*
Hypothetical	O’Connor et al.^21^ 2012	22471735	Addiction	USA	NH White NH Black Hispanic Other	471	14+
Hypothetical	Pearson et al.^22^ 2012	22994173	Am J Public Health	USA	NH White NH AA Hispanic Other	2649	18+
Hypothetical	Wackowski et al.^23^ 2014	24514070	Nicotine Tob Res	USA	White Black Hispanic Asian Other	2871	18–34
Hypothetical	Wackowski et al.^27^ 2015	25634935	Nicotine & Tobacco Research	USA	White Black Hispanic	519	18+
Hypothetical	D’Silva et al.^28^ 2015	-	Tobacco Regulatory Science	USA	NH White NH Black or AA Hispanic Other	9304	18+
Hypothetical	Harrell et al.^24^ 2017	28775996	Tob Regul Sci	USA	N/A	6809	12–17
Hypothetical	Zatoski et al.^18^ 2018	31516460	Tob Induc Dis	Non-USA	N/A	10760	18+
Actual Hypothetical	Chaiton et al.^10^ 2018	29507934	JAMA Intern Med	Non-USA	N/A	325	16+
Hypothetical	Pacek et al.^26^ 2019	30399498	Drug Alcohol Depend	USA	White Black Other	240	18–29
Hypothetical	Rose et al.^25^ 2019	31415195	Am J Public Health	USA		806	18–34
Actual	Chaiton et al.^11^ 2020	34350312	Tob Regul Sci	Non-USA	Non-White White	913	16+
Actual	Zatoski et al.^16^ 2020	32918816	Eur J Public Health	Non-USA	N/A	16534	18+
Actual Hypothetical	Chaiton et al.^12^ 2020	31147474	Tob Control	Non-USA	Non-White White	913	16+
Actual	Chaiton et al.^13^ 2021	33693745	Nicotine Tob Res	Non-USA	Non-White White	1821	16+
Hypothetical	Levy et al.^35^ 2021	34097061	Nicotine Tob Res	USA	N/A	n/a	35–54
Actual	Kyriakos et al.^17^ 2022	36163172	Tob Control	Non-USA	N/A	1326	18+
Hypothetical	Yang et al.^30^ 2022	35353183	Nicotine Tob Res	USA	White Black Asian Hispanic Other/mixed race	3248	18+
Actual	Chung-Hall et al.^14^ 2022	33820856	Tob Control	Non-USA	Non-White White	1236	18+
Hypothetical	Dearfield et al.^29^ 2022	35831050	BMJ Open	USA	Hispanic Non-Hispanic	221	18–80
Actual Hypothetical	Tam et al.^20^ 2024	38147008	Nicotine Tob Res	USA	Hispanic White Black Other	734	18–34
Hypothetical	White et al.^34^ 2023	36624010	Am J Prev Med	USA	African American/Black	579	18+
Hypothetical	Levy et al.^32^ 2023	34475258	Tob Control	USA	N/A	n/a	18–24
Actual	Fong et al.^15^ 2023	35483720	Tob Control	Non-USA	Black Non-Black	2320	18+
Actual Hypothetical	Booras et al.^19^ 2023	37239518	Int J Environ Res Public Health	USA	Black White Other	35	18+
Hypothetical	Yang et al.^31^ 2024	36446577	Tob Control	USA	N/A	3096	18+

* Those aged <18 years are considered adolescents.

**Table 2:** Summary rates of behavior transitions under actual and hypothetical menthol bans in US and non-US studies

*Ban*		*Quit menthol cigarettes*	*Switched to e-cigarettes*	*Continued menthol smoking*	*Switched to other combustibles*
		*Intended outcome*	*Harm reduction*	*Unintended consequences*	*Unintended consequences*
**Non-USA**					
**Actual**	Publications	8	4	5	4
	Rate, median (%)	22.0	33.5	27.0	34.1
	Min	11.0	29.1	14.1	22.8
	Max	29.1	28.0	51.6	59.1
**Hypothetical**	Publications	2	1	2	3
	Rate, median (%)	15.2	5.8	19.3	46.0
	Min	14.5	5.8	11.2	20.0
	Max	16.0	5.8	27.3	59.7
**USA**					
**Actual**	Publications	2	1	2	0
	Rate, median (%)	16.1	3.9	83.4	-
	Min	3.6	3.9	71.4	-
	Max	28.6	3.9	95.3	-
**Hypothetical**	Publications	16	9	8	9
	Rate, median (%)	29.0	21.2	24.9	40.7
	Min	7.0	12.3	6.7	12.5
	Max	65.7	48.8	60.0	59.1

### Non-US studies

*Actual ban*

Intended outcome: smoking cessation

The Canadian province of Nova Scotia made history by implementing the world’s first ban on menthol in tobacco products starting in 2015 continuing through 2017, with other Canadian provinces following suit by 2018. According to the first study of the actual response to the ban in Canada by Chaiton et al.^10^, 29.1% of menthol smokers quit smoking after 1 month of the ban. Similarly, they observed quit rates for daily or occasional menthol smokers at 24% or 20%, respectively, after 1 year of the ban^11,12^, but these rates were lower at 12.0% or 10.0% in the 2-year follow-up^13^. In a separate study conducted in Canada by Chung-Hall et al.^14,15^, higher quit rates for menthol smokers at 21.5% versus non-menthol smokers at 14.0% were observed after 2 years of menthol ban implementation (Table 1).

The European Union (EU) took the lead as the first major jurisdiction to introduce a ban on flavored cigarettes in 2016, including menthol as a ‘characterizing flavor’, not as an ingredient, which applies to all flavored cigarettes and roll-your-own (RYO) tobacco. The EU-wide ban on menthol cigarette sales went into effect in 2020 and has now included heated tobacco products (HTP) since 2022. Following a grace period, the 27 EU member states and the United Kingdom (UK) prohibited the sales of menthol cigarettes in 2020. Using survey data from eight EU countries, Zatoński et al.^16^ found that 14% of menthol smokers quit smoking after their access to menthol cigarettes was restricted. Interestingly for the Netherlands, Kyriakos et al.^17^ found that 66.9% of pre-ban menthol smokers attempted quitting, but only 17.8% and 26.1% of them succeeded in quitting smoking after 7 and 16 months of follow-up, respectively.

Harm reduction: switching to e-cigarettes

In the study of Chaiton et al.^10^, after one month following a menthol ban, they reported a larger proportion of menthol smokers (29.1%) using alternative flavored products, compared to the expected transition collected before the ban (5.8%), as menthol was not banned in e-cigarette products. Their 1-year follow-up study showed an even higher rate of those who switched to e-cigarettes at 42% for occasional and 34% among daily menthol smokers (38% on average)^11^. In summary, this longitudinal study showed an increased rate of transitioning to harm-reduction products, including e-cigarettes (29.1% after one month to 38.0% after one year following a menthol ban).

Unintended consequences: continued smoking

Chaiton et al.9 reported that 14.1% and 28.2% of menthol smokers continued menthol or non-menthol cigarette smoking only after one month of an actual menthol ban. Their later larger study (n=913) found 27.0% of pre-ban menthol smokers continued menthol smoking ^11^. In another study from Canada, Chung-Hall et al.^14^ reported that after the ban, 19.5% continued smoking menthol, while 59.1% of pre-ban menthol users switched to non-menthol cigarettes. From the study in the Netherlands, Kyriakos et al.^17^ found that 33.0% continued to smoke menthol cigarettes and 40.0% switched to non-menthol cigarettes. Zatoński et al.16 observed that 51.6% or 22.8% of pre-ban menthol smokers continued menthol smoking or switched to non-menthol cigarettes, respectively.

*Hypothetical ban*

Intended outcome: smoking cessation

Only a few non-US studies reported on the intention to quit under hypothetical bans. Zatoński et al.^18^ reported data from eight European countries, namely Germany, Greece, Hungary, Poland, Romania, Spain, England, and the Netherlands, right before the EU introduced the Tobacco Products Directive (TPD) in 2016, showing that 16.0% of menthol smokers (compared to 10.4% of other flavored cigarette smokers), aged ≥18 years, would quit smoking if menthol and flavored cigarettes were banned. Chaiton et al.^10^ showed that 14.5% of menthol smokers expected to quit before a ban.

Harm reduction: switching to e-cigarettes

There is only one non-US study reporting the intention to switch to alternative flavored products under a hypothetical ban. This suggests that there has been little attention to collecting data regarding expected behavior transitions to alternative products. Chaiton et al.^10^ reported that 5.8% of menthol smokers in Canada intended to switch to alternative flavored products, including e-cigarettes, cigars, and other flavored tobacco products, but this study did not provide e-cigarette-specific responses.

Unintended consequences: continued smoking

In Canada, Chaiton et al.^10^ predicted that 59.7%, 11.2%, and 1.9% of menthol smokers will use non-menthol cigarettes only, contraband menthol, or add menthol to their cigarettes, respectively. In another study^11^, the same group found that 46% of menthol smokers intended to switch to non-menthol cigarettes. In Europe, Zatoński et al.18 predicted that 27.3% or 20.0% of menthol cigarette users would find ways to obtain the banned product or switch to another brand (i.e. non-menthol, non-flavored), respectively.

### US studies

*Actual ban*

Intended outcome: smoking cessation

In the US, during the same timeframe, two states (California and Massachusetts) and 170 localities banned menthol cigarettes, and there are only two studies (both from Massachusetts) regarding behavior changes after actual menthol bans in the US. Booras et al.^19^ studied the impact of the menthol ban on those who had at least one counseling session with a tobacco treatment specialist (aged ≥18 years). They reported that 28.6% of menthol smokers quit smoking completely 6 months post-ban in Massachusetts on a small sample size (n=14), slightly lower than anticipated pre-ban behavior (36.0%). A much lower quit rate was found in a bigger sample size of young adult menthol smokers (n=734), with oversampling in Massachusetts by Tam et al.^20^. This study showed that 3.6% of pre-ban exclusive menthol smokers and 9.0% of those who used both e-cigarettes and cigarettes were able to quit smoking in response to the ban. These numbers were notably lower than the rates of intentions to quit under a hypothetical ban (29.6% and 12.4%, respectively) reported in the same study.

Harm reduction: switching to e-cigarettes

Of the two US studies conducted in Massachusetts, Tam et al.^20^ found that 3.9% of exclusive smokers reported using e-cigarettes after the ban, which is much lower than the rate of dual users (43.7%). However, Booras et al.^19^ did not capture menthol smokers’ transition to e-cigarettes.

Unintended consequences: continued smoking

In the US (i.e. Massachusetts), Tam et al.^20^ found that after two years of the actual ban, most exclusive menthol cigarette users and dual users with e-cigarettes continued to smoke menthol cigarettes by obtaining them from an alternate source (95.3% and 86.9%, respectively). A longitudinal study conducted in Massachusetts by Booras et al.^19^ reported that 71.4% (10/14) of pre-ban menthol smokers continued to smoke menthol cigarettes, including 43% less, 21% same, and 7% more than pre-ban use.

*Hypothetical ban*

Intended outcome: smoking cessation

Several studies assessed tobacco use behavior under the hypothetical ban in the US shortly after the signing of the Tobacco Control Act in 2009. Using data collected from the 2010 Current Population Survey Tobacco Use Supplement, O’Connor et al.^21^ found that 35.0% of menthol smokers aged ≥14 years would attempt to quit if menthol cigarettes were banned. Non-Hispanic Blacks (43.8%) had a slightly higher quit intention compared to non-Hispanic whites (35.2%). A similar intended quit rate of 44.5% among non-Hispanic African Americans, slightly higher than the general population, was found by another study^22^.

A study by Wackowski et al.^23^ using data from the 2011 National Young Adult Health Survey showed a higher expected quit rate of 65.7% among young menthol smokers (aged 18–34 years). A study by Harrell et al.^24^ of the two large cross-sectional surveys from the Texas Adolescent Tobacco and Marketing Surveillance System, adolescents (aged 12–17 years) and young adults (aged 18–29 years) from the Marketing and Promotions across Colleges in Texas reported that 53.9% and 44.2% of menthol cigarette users would not use the product if it were not flavored, respectively^24^. However, the intended quit rates seem to be lower for young adult menthol smokers (23.5%), with similar responses over time^25^, as reflected in longitudinal data (2011–2016) from the National Young Adult Health Survey (NYAHS). Paceck et al.^26^ also found approximately the same rate at 25% for young adults.

For adult menthol smokers, the intended quit rate was only 28.4%^27^. This is reflected in the discrepancy between older adults aged ≥45 years and younger smokers who reported intent to quit (40.1% vs 20%, respectively)^27^. From the 2014 Minnesota Adult Tobacco Survey, D’Silva et al.^28^ reported that 46.4% of menthol smokers responded with the intention to quit smoking if menthol cigarettes were no longer sold in the US. A similar rate of 48.0% was found by Dearfield et al.^29^, which focused on low socioeconomic status residents aged 18–80 years in the District of Columbia (DC) who smoke menthol cigarettes (83.3% African Americans/Black). In a recent study of menthol smokers aged ≥18 years by Booras et al. ^19^, 35.7% anticipated to continue menthol cigarettes. However, a much lower rate was found by Yang et al.^30^ when they assessed how smokers using menthol cigarettes, flavored cigars, or e-cigarettes would respond to three different flavor ban scenarios that include all three products. They found that exclusive menthol cigarette users were more likely to quit all tobacco use in the event of a menthol ban (7%), higher than those who use both menthol cigarettes and flavored cigars (0.3%) and those who smoke flavored cigars only (0.9%). Yang et al.^31^ assessed how smokers using menthol cigarettes, flavored cigars, or e-cigarettes would respond to three different flavor ban scenarios that include all three products. They found that banning menthol cigarettes and flavored cigars would yield a 12.6–20.5% quit rate, regardless of whether menthol-flavored e-cigarettes were still available. Levy et al.^32^ predicted similar quit rates between those aged 18–24 years (17.7%) and 35–54 years (14.7%). Interestingly, they also predicted a 15.0% decrease in overall smoking by 2026, which is much lower than their previous study^33^, showing a 35.7% decrease by the same year. White et al.^34^ found that African American menthol smokers were more likely to quit under a comprehensive (ban characterizing flavors in all tobacco products) or cigarette-only ban (54.1%) compared to the status quo (43.5%). Using expert elicitation and data from the PATH study, Levy et al.^35^ predicted that the average quit rate for the general population is 21.7% or 22.5% for age groups 18–24 or 35–54 years, respectively, which were comparable to the rate for African American menthol smokers (25.2% or 27.8%).

Harm reduction: switching to e-cigarettes

D’Silva et al.^28^ reported that 12.3% of menthol smokers would switch to e-cigarettes in response to a proposed ban. A similar rate for anticipated switching to menthol e-cigarettes was reported by Wickham^36^ at 15.1%, with a higher percentage of switching among Black (23.0%) and White (18.3%) compared to Hispanic menthol smokers (0.7%). The overall similar switching rate to menthol e-cigarettes at 13.0% was observed in a later study by Dearfield et al.^29^. However, Yang et al.^30^ found that 25.6% of menthol smokers would switch to e-cigarettes. They found that using menthol cigarettes regularly or occasionally did not affect the decision to switch to e-cigarettes in response to a menthol ban.

Experts in the study by Levy et al.^35^ predicted a higher intended switching rate to novel nicotine delivery products (NNDPs), including e-cigarettes, at 24.1% and 20.0% of menthol smokers aged 18–24 and 35–54 years, respectively, compared to the status quo (no menthol ban, 8.5%, and 9.7%, respectively). Their data show that African American menthol smokers seem less likely to switch to NNDPs at 21.6% and 17.0%, respectively. These numbers are similar to what they reported in a later study^32^. White et al.^34^ reported more menthol smokers anticipated switching to e-cigarettes under a limited ban (flavors in cigarettes and cigars) at 48.4% compared to a comprehensive ban (flavors in all tobacco products) at 42.2%, and both higher than the status quo 36.9%. Under three different ban scenarios, Yang et al.^31^ found the lowest switching intention to e-cigarettes if all flavor tobacco products are banned (20.8%) compared to limited flavor bans (33.3–38.8%). Tam et al.^20^ found that young adult users of both menthol cigarettes and e-cigarettes are more likely to switch completely to e-cigarettes (41.4%) in response to a ban, compared to those who exclusively use cigarettes (14.7%).

Unintended consequences: continued smoking

In the US, an early study by O’Connor et al.^21^ showed that 25.0% of menthol smokers aged 14–26 years would find a way to buy a menthol brand. Studies of young adult menthol smokers showed that 12.5%^22^, 18.4%^23,27^, or 32.3%^25^ of menthol smokers anticipate switching to non-menthol cigarettes in response to a menthol ban. D’Silva et al.^28^ found that 53.6% and 26.6% of older adults would continue to smoke menthol or switch to non-menthol cigarettes, respectively. Another study by Dearfield et al.^29^ found a different inverse pattern for continuing to use menthol cigarettes (24.9%) or switching to non-menthol products (40.7%) under a hypothetical menthol ban. Similarly, Yang et al.^30^ found that 17% or 53.6% of exclusive menthol smokers would continue using menthol cigarettes or switch to unflavored cigarettes, with Black smokers being less likely to switch to nonflavored smoking compared to White smokers (OR=0.69).

Under different policy scenarios, Levy et al.^35^ found that compared to the status quo (no ban, 70.2% vs 79.6 for the general population and African Americans only, aged 18–24 years, respectively), under a menthol ban, substantially lower rates were found for switching to non-menthol cigarettes (40.3% vs 35.1%, respectively) and for continuing menthol cigarette or cigars (6.5% vs 7.6%, respectively). These rates were comparable for older menthol smokers aged 35–54 years. Yang et al.^31^ found that menthol smokers had similar rates of intention to switch to non-menthol cigarettes or cigars under three different ban scenarios (46.3–51.6%), which are slightly lower among dual users with e-cigarettes (32.7–41.3%). Their later report predicted that 10.1% or 8.8% would continue to smoke menthol cigarettes and 24.4% or 59.1% would switch to non-menthol cigarettes among current menthol smokers aged 18–24 or 35–54 years, respectively^32^. Similarly, White et al.^34^ found that 41.8% or 42.4% of African American menthol smokers would try to buy or import menthol cigarettes from unlicensed retailers’, under limited (cigarettes and cigars) or comprehensive (all tobacco products) flavor bans, respectively, which are higher than status quo (no ban, 33.3%).

Booras et al.^19^ reported that 60% of menthol smokers would use menthol cigarettes less or not change how much they smoke menthol cigarettes. Tam et al.^20^ online survey of young adults aged 18–34 years found that the majority of those who exclusively smoke menthol cigarettes and who dual use with e-cigarettes predicted they would continue smoking (72.2% vs 71.8%).

## DISCUSSION

Understanding the behavioral transitions of menthol smokers in response to a menthol ban has important implications for public health and tobacco control. This scoping review summarizes the up-to-date literature, including the most recent publications on behavior transitions following actual menthol bans in the US (state level).

The ultimate goal of a policy that restricts the sale and access to menthol cigarettes is to support individuals who smoke menthol cigarettes to quit tobacco use (intended outcome). However, due to the highly addictive nature of nicotine and the synergistic effects of menthol on this addiction^36,37^ , some menthol smokers may turn to alternative tobacco products (e.g. e-cigarettes) that deliver nicotine and menthol while maintaining a lower risk profile (harm reduction). Unfortunately, others may switch to non-menthol cigarettes or other combustible products or obtain their menthol cigarettes from illicit sources (unintended consequences), posing potential risks to public health, as summarized in this scoping review.

### Geographical/historical diversity

We found more studies in the US focused on hypothetical bans, while non-US studies reported data from actual bans. This can be attributed to Canada and Europe implementing national bans in 2017^38^ and 2020^39^, respectively. While the US does not have a federal ban yet, there have been state-level menthol (or flavor) bans in Massachusetts in 2020^40^ and California in 2022^41^, as of January 2024. The number of publications regarding actual and hypothetical bans indicates that US researchers have proactively tried to understand the impact of a federal menthol ban compared to their non-US counterparts. Under actual menthol bans, in two studies^19,20^ conducted in the US, the rate of continued use of menthol cigarettes after a state ban (i.e. Massachusetts) was found to range from 71.4% to 95.4%. This range is more than double the predicted rate of 29.6–36.0% reported in the same geographical locations. This discrepancy highlights the need for effective cessation support interventions. Further, these data support an urgent need for a federal menthol ban, as national menthol bans are more effective than local, or state menthol bans^6^.

When behavior transition under the actual menthol ban in Massachusetts was compared with predicted scenarios from national data, the rate of switching to flavored alternative tobacco products, including e-cigarettes, seems generally overestimated compared to the rates under the actual bans (21.2% vs 3.9%). Conversely, the opposite pattern was found in non-US studies (5.8% vs 29.1%). Given the potential lack of generalizability of state- or country-specific data, it is important to conduct further research on the differences and similarities in behavioral scenarios with both hypothetical and actual bans at the state and federal levels. This research will help maximize reductions in smoking by promoting cessation programs and implementing rigorous communication strategies for these programs at the state and federal levels. Also, considering the geographical diversity of menthol smokers in the US^42^, with 73% of African American menthol smokers historically targeted by tobacco industries, and this population is regionally concentrated in the Southern US (56%), additional studies, particularly from the US population under actual menthol bans, will be needed.

### Age-specific considerations

There are unique vulnerabilities and challenges associated with tobacco use across different age groups, and menthol cigarettes are particularly prevalent among young individuals^43,44^. Therefore, it is important to examine the variations or similarities in behavioral transitions in response to a menthol ban among different age groups. However, two recent US studies under the actual ban did not include adolescents^19,20^. This is an important research gap, as menthol cigarettes appeal more to youth due to their milder taste and are perceived as less harmful relative to non-menthol cigarettes^45,46^.

While over the two decades, cigarette sales have declined significantly, the majority of the decline is attributed to declines in non-menthol cigarettes (by 53% from 2000 to 2018 vs just 26% for menthol cigarettes)^47^. According to nationally representative data from the National Survey on Drug Use and Health (NSDUH) in 2018^1^, 49.7% of cigarette smokers aged 12–17 years preferred menthol compared to 38.7% of those aged 35–49 years, 33.1% of those aged 50–64 years, and 29.1% of those aged ≥65 years. Thus, we recommend further studies to track trajectories of smoking behaviors following menthol bans, especially among youth. These studies should also explore youth perception of menthol bans and awareness of freely available cessation tools and support, such as Quitline.

### Racial and ethnic disparities

As it is well documented, menthol cigarette smoking is disproportionately prevalent among African Americans/Blacks (85% vs White 30%) in the US^1^, mainly due to the tobacco industry’s predatory marketing in Black communities^48^. Thus, a menthol ban could benefit African American/Black menthol smokers if they quit smoking^32,33,49,50^. As of today, as presented here and by others, most studies on the effects of actual menthol bans are based on non-US populations with substantially different racial and ethnic characteristics of menthol smokers as well as different social determinants of health^51^. When it comes to the US, two studies under actual bans did not provide stratified rates by ethnic/race groups^19,20^. Thus, it is critical to collect historical data before and after a federal menthol ban, particularly among those historically targeted by the tobacco industry.

### Recommendations for future research

This scoping review summarized the rates of behavior transitions leading to intended and unintended outcomes, as well as harm reduction in actual and hypothetical menthol bans among US and non-US populations. Given the lower rate of intended outcomes (quitting smoking) of state-level menthol bans from the recent US studies, a federal ban on menthol cigarettes would be more effective in benefiting public health in the US. Additionally, after the implementation of actual menthol bans, longitudinal analyses of behavior transitions have shown reduced smoking quit rates over time. This indicates a failure in long-term cessation, highlighting the need for further research in developing and promoting effective and accessible cessation programs for those who have attempted or intended to quit. The main unintended consequences of a US federal menthol ban are continued smoking of menthol cigarettes, switching to non-menthol cigarettes or other combustible tobacco products, and the disproportionate impact of these consequences on population subgroups. Data from other jurisdictions showed that continued smoking after the ban could limit its public health benefit if the policy is not supported by cessation intervention and educational campaigns. Additionally, more data under different policy scenarios for exclusive menthol smokers and dual users of cigarettes and e-cigarettes are needed. In addition, further studies focusing on the benefits of banning other ‘modified’ tobacco products, such as heavily filtered cigarettes marketed as ‘light’, low’, and ‘mild’ cigarettes, are needed. Similar to menthol cigarettes, these products are designed to make smoking more appealing and contribute to harm misperceptions^52^. Thus, banning these products could help to maximize smoking cessation. Additional suggested future research is to explore the potential impact of illegal/black-market tobacco on a national menthol cigarette ban. For example, in Australia, the strong public health initiative aimed at eliminating smoking has led to a significant increase in black-market tobacco, often sold at prices lower than retail^53^. This situation is keeping ‘hardcore’ smokers from continuing their habits longer while also making it easier for young people and new smokers to access tobacco – an opportunity that would have been much more challenging and costly just a decade ago^53^. Thus, the FDA needs more information on what policy scenarios would be most effective for menthol smokers to quit smoking and what e-cigarette characteristics are attractive to individuals looking to switch.

### Limitations

While our scoping review provides an up-to-date study summary regarding behavior transitions under hypothetical and actual bans in the US and non-US studies, it is important to note limitations. Because of a wide range of study designs and data collections across publications, our summary was limited to presenting a rate summary range of transitions to the different scenarios without judging the study design, characteristics of study populations, or sample size. Additionally, the quality of summarized studies and the used methodologies (i.e. online or in-person surveys) were not evaluated as part of this scoping review. Due to a small number of US studies under actual bans (n=2), the transition rates presented cannot be generalizable to the US population, emphasizing the need for future studies. Furthermore, the database PubMed was used for this review in addition to tracking forward citations of recent review articles, which may not cover other references related to the topic of this review.

## CONCLUSIONS

We have identified research gaps in this topic regarding geographical coverage, age-specific considerations, racial/ethnic representation, and general research focuses to be captured. Our recommendations can help prioritize future research focusing on cultural, social, and economic factors to address the unique needs and challenges of diverse population groups in the US following a federal menthol ban. Ultimately, prioritizing these areas will help policymakers understand the dynamic changes (both short- and long-term) in smoking behaviors after a federal ban and further promote public health by considering the diverse support needs for smoking cessation and perspectives of communities in the US.

## Data Availability

Data sharing is not applicable to this article as no new data were created.
